# Psychological well-being of healthcare workers during COVID-19 in a mental health institution

**DOI:** 10.1371/journal.pone.0300329

**Published:** 2024-03-18

**Authors:** HoiTing Leung, Madeline Lim, Wee Onn Lim, Sara-Ann Lee, Jimmy Lee

**Affiliations:** 1 Department of Psychology, Institute of Mental Health, Singapore, Singapore; 2 Research Division, Institute of Mental Health, Singapore, Singapore; 3 Population and Global Health, Lee Kong Chian School of Medicine, Nanyang Technological University, Singapore, Singapore; 4 North Region, Institute of Mental Health, Singapore, Singapore; National Cheng Kung University College of Medicine, TAIWAN

## Abstract

**Introduction:**

This study examined the psychological wellbeing of Healthcare Workers (HCWs) during COVID-19 in a mental health setting, associations of psychosocial wellbeing with coping style, and ways that organisations can mitigate the psychosocial burden on HCWs.

**Methods:**

Thirty-seven Mental HCWs (MHCWs) from infected and non-infected wards (control group), were recruited and assessed at three timepoints. Psychological wellbeing, perceived cohesion, and coping style (Brief-COPE) were assessed. Reports on individual coping and feedback on the organisation were collected through in-depth interview. Comparison between infected and non-infected wards, as well as comparison of psychosocial measures and perceived cohesion, across the three timepoints were made. As there were no significant changes in coping styles across the timepoints, Timepoint 1 (T1) coping style was used to correlate with the psychosocial measures across all timepoints. Thematic analysis was used for qualitative data.

**Results:**

MHCWs from infected wards reported significantly higher levels of stress, χ^2^(1) = 6.74, p = 0.009, effect size: medium (ε^2^ = 0.198), and more severe sleep disturbance (PSQI), χ^2^(1) = 6.20, p = 0.013, effect size: medium (ε^2^ = 0.182), as compared to the control group at T2. They also engaged in more problem-focused coping (T2 and T3) and emotion-focused coping (T2). As expected, negative coping style was correlated with negative outcomes except problem-focused coping that was correlated with both negative (sleep disturbance and anxiety symptoms) and positive outcomes (wellbeing). Emotion-focused coping was moderately correlated (T_b_ = 0.348, p<0.017) with higher levels of wellbeing at T2. Thematic analyses revealed MHCWs felt supported by the responsiveness of the institution, emotional and informational support, and the availability from direct leaders, presence of team and hospital leaders on the ground, helped build trust and confidence in the leadership.

**Conclusions:**

MHCWs experienced significantly higher levels of stress and sleep disturbance during COVID-19. The ways that organizations can offset the psychological burden of pandemics on MHCWs are discussed.

## Introduction

Infectious disease outbreaks bring about negative psychological impact on healthcare workers (HCWs) [1–4] and the coronavirus disease 2019 (COVID-19) pandemic was no exception [3, 5–8]. Disease outbreaks can be exceptionally challenging for HCWs as they struggle to cope on both work and personal levels. HCWs must balance between their care duties and personal needs of safety, in order to keep family, and friends safe from infections [9]. COVID-19 was especially stressful as there were no vaccines and definitive treatment for it during the early phases of the pandemic, and the only protection that HCWs could turn to were nonpharmaceutical interventions, such as the full personal protective equipment (PPE) and social distancing [10]. Other stressors included false claims and misinformation spreading via social media platforms [11, 12], as well as changing work roles due to new work arrangements aimed at curbing disease transmissions. These exacerbated the stress that HCWs were already facing [3, 5–7].

It is crucial to examine HCWs’ psychological well-being and inform the development of interventions to mitigate expected poor outcomes [8] during a novel disease outbreak as prolonged exposure to stress can result in burnout [13] and preventing high staff turnover rate is paramount as healthcare systems are primarily sustained by HCWs. Publications on the impact of COVID-19 on physical and psychological health of HCWs in acute medical and psychiatric settings have springed up since the onset of the pandemic [3, 5, 6, 9, 14]. Additional challenges and complexities in caring for people with mental health conditions during the COVID-19 pandemic have been highlighted in some of these literatures.

In psychiatric hospitals, patients reside in closer proximity, interact more closely and frequently with nursing staff, and use more communal spaces such as the bathrooms and dining room. They also have higher rates of physical health comorbidities which increases the risk of COVID-19 infection and are associated with poorer outcomes if infected [15, 16]. They may exhibit irrational behaviours, anger and impulsivity, and it may be difficult for them to adhere to infection control measures such as hand washing and mask-wearing [17]. Social distancing and restricted social visitations prevent psychiatric patients from receiving social interaction as a form of therapy since many communal activities like dining, watching television together, group meetings and activities are vital sources of emotional and spiritual support for people struggling to stay in recovery [16, 18]. Such prolonged isolation, disruption to services, coupled with the fear of getting infected by a possibly life-threatening virus, as well as the ever changing and uncertain pandemic-related information, can trigger and exacerbate more anxiety and depressive symptoms [18, 19]. The challenge is added with elderly and children that have neurocognitive diagnosis such as dementia [20], Autism Spectrum Condition (ASC) and Attention Deficit Hyperactivity Disorder (ADHD) [21], respectively. There may be differential impact of the pandemic across the psychiatric professions, but nurses have been identified to have suffered the most workload, since they had to take on more infection control and screening tasks, consequently also reducing the interactional part of their work [22].

Since previous publications were cross-sectional and descriptive in nature, examining the psychological wellbeing of HCWs at a specific time point, this research seeks to examine the psychosocial impact of COVID-19 in a mental health setting using a longitudinal approach through the Singapore sample as a sole focus on the mental health setting is still lacking in this sample [8, 23–25]. Singapore confirmed its first case of COVID-19 in January 2020 and experienced a peak in daily imported cases in March 2020 [24]. A nation-wide lockdown was instituted between April 7 to June 1 [26] and healthcare systems had to adjust to constant changing protocols to cope with the pandemic [27].

The current study examined the psychosocial wellbeing of MHCWs over a period of less than 2 years at a psychiatric institute in Singapore. Wards that did not experience COVID-19 infection were used as a control group for comparison as no data prior to the pandemic was available. In addition, this study seeks to examine the relationship between individual coping strategies and psychosocial wellbeing as no study has done so. We hypothesized that (i) MHCWs from wards with COVID-19 infection would experience greater psychological distress and (ii) MHCWs who engaged in positive coping styles and/or perceived greater team cohesion would experience less psychological distress. Burnout in healthcare workers has received increased attention due to the COVID-10 pandemic, with advocates rallying for more research and organizational interventions to mitigate the impact of staff burnout on quality healthcare [30]. In line with this advocacy, this research sought to identify both individual and organizational interventions which HCWs in a psychiatric setting have found helpful during the COVID-19 pandemic. These could provide applied interventions for future references, in the context of the psychiatric setting.

## Materials and methods

### Setting

In April 2020, the first COVID-19 infection was confirmed amongst inpatients at a psychiatric institute in Singapore. Proactive swab tests were conducted for all staff and patients who had been in the ward during the at-risk period following contact tracing. This move detected four more inpatients with COVID-19. Staff continued to nurse the inpatients under quarantine while awaiting swab results. A ward was also immediately converted into a COVID-19 isolation ward, housing inpatients who were diagnosed with COVID-19. This also coincided with the circuit breaker period in Singapore [26].

### Study design

This mixed-methods study consists of a longitudinal survey and an in-depth interview. Recruitment emails were sent to the three inpatient wards that had contact with COVID-19 cases and three wards that had no contact as controls. Interested participants either liaised with the ward clinicians or study team. Only staff who had been working on a full-time basis during the COVID-19 period and had not taken any leave (including maternity) were included in the study. Ethics approval for this study was granted from the National Healthcare Group’s Domain-Specific Review Board (Approval No. 2020/00969) in accordance with the ethical standards laid down in the 1964 Declaration of Helsinki and its later amendments.

The longitudinal survey took place over three time points, conducted via online questionnaire platform SurveyMonkey. Timepoint 1 (T1) was from June to August 2020 when strict workplace measures such as leave restrictions, lunching alone, and adjustments to provide services remotely were implemented. However, a retrospective method was used as study conceptualisation and ethics approval led to delay. Participants were asked to complete the survey by recall. To ease the recall, an excerpt of the institution’s Chief Executive Officer (CEO) message about the detection of the first COVID-19 case was attached at the beginning of the survey at T1. Timepoint 2 (T2) was conducted from January to June 2021 when the social distancing measures (SDM) were relaxed to allow lunch with another colleague from the same team and taking of leave albeit with restrictions. Timepoint 3 (T3) was conducted from April to June 2022 when SDM eased further to allow lunches with more than one other colleague, taking of leave without restrictions, and resumption of face-to-face meetings. The in-depth interview was conducted in groups and was made optional via video conferencing after T2 survey completion.

The survey comprised of self-report measures listed below. In-depth interview questions included participants’ responses when they first became aware of COVID-19, their response when the first confirmed case was reported in the current hospital, how did they and their team cope, what were the areas done well by the team or hospital management, and their feedback on areas for improvement. All participants received an explanation of the study’s aim and provided written indication of informed consent prior to the baseline assessment.

### Instruments

#### Depression Anxiety and Stress Scale-21 (DASS-21)

The DASS-21 is a 21-item measure that assesses depression, anxiety, and stress symptoms, rated on a four-point Likert scale. Cut-off scores of more than 9, 7 and 14 indicate a positive screen for depression, anxiety, and stress respectively [28]. DASS-21 has been tested on clinical [29] and community samples [30] where it has shown to be able to distinguish depression, anxiety and stress with acceptable to excellent ranges of internal consistency and concurrent validity. It is also been tested valid in Asian community samples [31].

#### Short Warwick Edinburgh Mental Well-Being Scale (SWEMWBS)

The SWEMWBS [32] comprises seven statements about individuals’ mental wellbeing over the last two weeks, rated on a scale of 1 (none of the time) to 5 (most of the time). Total scores < 20 were interpreted as low well-being, 20–27 as moderate well-being and > 27 as high well-being. This interpretation was provided by the scale developers’ excel template for WEMWBS 7-item calculation. The 7-item SWEMWBS has been validated in a sample of clinical population in Singapore and has shown adequate psychometric properties to be used [33].

### Pittsburgh Sleep Quality Index (PSQI)

The PSQI is a 19-item measure assessing sleep quality [34], comprising of seven categories. Participants rate their sleep experiences over the past month on a scale of 0 (not for the past month) to 3 (three or more times a week), with higher scores indicating more sleep disturbances. Total scores of ≤ 5 is interpreted as good sleep quality and > 5 as poor sleep quality. The PSQI has shown adequate psychometric properties in clinical and non-clinical samples [35], including a local sample [36], as a screening tool for insomnia [37].

#### Perceived Cohesion Scale (PCS)

The PCS is a six-item questionnaire measuring perceived group cohesion by assessing individuals’ sense of belonging and feelings of morale [38]. Participants rate on a scale of 0 (strongly disagree) to 6 (strongly agree). Total scores were averaged, with higher scores indicating higher perceived cohesion. The PCS has been tested to be valid and reliable in small groups [39].

#### Brief-COPE

The Brief-COPE [40] is a 28-item questionnaire assessing participants’ coping strategies on a four-point Likert scale from 1 (I haven’t been doing this at all) to 4 (I’ve been doing this a lot). There are 3 overarching coping styles: problem-focused coping, emotion-focused coping, and avoidant coping. Scores were interpreted by using the mode score per item, at each time point before entering into NovoPsych [41] which synthesizes and presents the normative percentile and clinical percentile scores. Although some studies have found that the two-factor second-order model is generally a good fit [42, 43], the three-factor model will be used in this study as NovoPsych has used data from studies [44, 45] that have established the three-factor model, with adequate psychometric properties, for norming.

### Data analysis

#### Quantitative analysis

Comparison between the infected wards (target group) and control wards (control group) were made using Kruskal Wallis test as the data was non-normal. Comparisons were made for all three visits and effect size of the difference was calculated due to the small sample size. Epsilon square (ε^2^) was used to quantify the effect size and the interpretation of the effect sizes were interpreted as the following: < 0.02 (very small); < 0.13 (small); < 0.26 (medium); ≥ 0.26 (large) [46]. Descriptive summaries of the psychosocial measures and perceived cohesion measure across the three time points were also analysed and compared. For comparison analysis across the three time points, one-way repeated measures ANOVA test was used unless the data was non-normal. The Friedman one-way repeated measure of analysis of variance by ranks test (Friedman test) was used as the non-parametric test.

As part of the longitudinal analysis, coping styles at each of the three time points were compared to identify if there were any change in coping styles across the time points. Kruskal-Wallis tests was used as the data was non-normal. As there were no significant changes in coping styles across the timepoints, Timepoint 1 (T1) coping style was used to correlate with the psychosocial measures across all timepoints. This method of analysis allows more causal explanation as while coping style is held constant, any change in its association with the psychosocial variables across time could be said to be a result of its effect on the psychosocial variables.

Kendall Tau-B correlation was used for the association between T1 coping style with T1 to T3 psychosocial variables as the data was non-normal. The strength of association was determined by the criteria of 0.1 to 0.3 as small correlation, 0.3 to 0.5 as moderate correlation, ≥ 0.5 as large correlation [47]. Other reasons Kendall Tau-B was used is because it has been recommended for smaller sample size data [4] and is more conservative than Spearman Rho [48, 49]. All tests were conducted on IBM SPSS (version 23). Statistical significance was set at p <0.017 after Bonferroni correction (0.05/3) was applied as multiple comparison was involved.

#### Qualitative analysis

Thematic analysis based on the six phases as recommended by Braun and Clarke [50] was adopted to draw common themes of experiences shared (inductive approach). The six phases includes: familiarising with data, generating initial codes, searching for themes, reviewing themes, defining and naming themes, producing the report. No software was used for the qualitative analysis, all analyses were conducted and compiled using Microsoft Excel, and circulated amongst the first three authors for discussion and concurrence.

In accordance to Braun and Clarke [50], the transcripts were read and re-read by the first author to sort the responses into preliminary codes before assigning them into categories, and grouped into broader domains (themes). It was then passed on to the second author for review, who then passed to the third author for further review and agreement (reviewing). A meeting was conducted between first three authors to discuss on the coding, and eventual definition of themes and labelling for the data (refining).

## Results

### Sample characteristics

The characteristics of participants are summarised in Table 1. A total number of 37 responses were collected for T1, but due to dropouts across the time points, there were 35 responses for T2 and 15 responses for T3. Attrition analysis was conducted (see S1 & S2 Tables). We found that there were no significant differences between participants that dropped out of the study at T3 and those that stayed on, except that participants whom dropped out used more avoidant coping (*X*^2^ = 7.74, p = 0.005)- denial in particular (*X*^2^ = 5.78, p = 0.016). Across the three timepoints, there were almost equal distributions of gender except in T3 where there were more females. Demographics of the participants are presented in Table 1. There were no significant differences in sociodemographic factors between the MHCWs in the infected group (staff working in wards with infected case or cases) and the control group.

**Table 1:** Characteristics of study sample across time points.

Characteristic	T1 (n = 37)	T2 (n = 35)	T3 (n = 15)	Infected group (n = 18)	Control group (n = 19)	*X* ^2^	p-value
**Gender, *N (%)***						1.337	0.330
Male	19 (48.6)	18 (51.4)	5 (33.3)	11 (61.1)	8 (42.1)		
Female	18 (51.4)	17 (48.6)	10 (66.7)	7 (38.9)	11 (57.9)		
**Age range, N (%)**						3.045	0.385
20–30	10 (27.0)	9 (25.7)	2 (13.3)	7 (38.9)	3 (15.8)		
31–40	15 (40.5)	14 (40.0)	8 (53.3)	7 (38.9)	8 (42.1)		
41–50	6 (16.2)	6 (17.1)	2 (13.3)	2 (11.1)	4 (21.1)		
51–60	6 (16.2)	6 (17.2)	3 (20.0)	2 (11.1)	4 (21.1)		
**Ethnicity, *N (%)***						3.994	0.262
Chinese	14 (37.8)	13 (37.1)	7 (46.7)	6 (33.3)	8 (42.1)		
Malay	7 (18.9)	7 (20.0)	3 (20.0)	3 (27.8)	2 (10.5)		
Indian	4 (10.8)	4 (11.4)	1 (6.7)	5 (16.7)	1 (5.3)		
Others	12 (32.4)	11 (31.4)	4 (26.7)	4 (22.2)	8 (42.1)		
**Marital status, *N (%)***						2.450	0.294
Single	14 (37.8)	13 (37.1)	5 (33.3)	8 (44.4)	6 (31.6)		
Married/living with someone as if married	23 (62.2)	22 (62.9)	10 (66.7)	10 (55.6)	13 (68.4)		
**Children, N (%)**						3.246	0.103
Yes	20 (54.1)	19 (54.3)	9 (60.0)	7 (38.9)	13 (31.6)		
No	17 (45.9)	16 (45.7)	6 (40.0)	11 (61.1)	6 (68.4)		
**Years in Service, N (%)**						4.955	0.084
0–5 years	13 (35.1)	12 (34.3)	5 (33.3)	9 (50.0)	4 (21.1)		
6-15 years	15 (40.5)	15 (42.8)	6 (40)	7 (38.9)	8 (42.1)		
>15 years	9 (24.4)	8 (22.9)	4 (26.7)	2 (11.1)	7 (36.8)		
**Job Designation, N (%)**						6.981	0.073
Managerial Nurse	10 (27.0)	10 (29.0)	6 (40.2)	3 (16.7)	9 (47.4)		
Staff Nurse	19 (51.3)	17 (49.3)	7 (46.9)	14 (77.8)	9 (47.4)		
Assistant Nurse	6 (16.2)	6 (17.4)	2 (13.4)	1 (5.6)	0 (0.0)		
Psychiatrist	1 (2.7)	1 (2.9)	0 (0.0)	0 (0.0)	1 (5.3)		
Peer Support Specialist	1 (2.7)	1 (2.9)	0 (0.0)	3 (16.7)	9 (47.4)		
**Chronic Physical Condition, N (%)**						0.173	1.000
Yes	5 (13.5)	5 (14.3)	2 (13.3)	2 (11.1)	3 (15.8)		
No	32 (86.5)	30 (85.7)	13 (86.7)	16 (88.9)	16 (84.2)		

Number of Children: N = 19 (T1), 18 (T2), 9 (T3)

### Comparison of psychosocial measures between infected group and control group across timepoints

There were no differences between the infected and control ward groups in psychosocial measures for T1 and T3 (see S3 & S4 Tables). Differences were found between the two groups in T2 and reported in Table 2.

**Table 2:** Descriptive statistics of measures- comparison between infected and control group for Visit 2.

Measure	Total	Infected group	Control group	*X* ^2^	p-value
	(n = 35)	(n = 18)	(n = 17)		
**DASS-21 Stress, *Mean (SD)***	7.54 (6.36)	10.11 (6.81)	4.82 (4.64)	**6.74**	**0.009**
Normal range, *N (%)*	33 (94.3)	16 (88.9)	17 (100.0)		
Positive range, *N (%)*	2 (5.7)	2 (11.2)	0 (0.00)		
**DASS-21 Anxiety, *Mean (SD)***	4.46 (4.65)	6.00 (5.22)	2.82 (3.40)	3.32	0.068
Normal range, *N (%)*	26 (74.3)	11 (61.1)	15 (88.2)		
Positive range, *N (%)*	9 (25.7)	7 (38.9)	2 (11.8)		
**DASS-21 Depression, *Mean (SD)***	5.83 (5.52)	7.22 (6.06)	4.35 (4.60)	1.74	0.187
Normal range, *N (%)*	26 (74.3)	11 (61.1)	15 (88.2)		
Positive range, *N (%)*	9 (25.7)	7 (38.9)	2 (11.8)		
**SWEMWBS, *Mean (SD)***	23.87 (3.69)	24.46 (4.28)	23.24 (2.95)	0.83	0.361
Low, *N (%)*	5 (14.3)	3 (16.7)	2 (11.8)		
Moderate, *N (%)*	27 (77.1)	12 (66.7)	15 (88.2)		
High, *N (%)*	3 (8.6)	3 (16.7)	0 (0.0)		
^**a**^ **PSQI, *Mean (SD)***	6.82 (3.60)	7.88 (3.82)	5.76 (3.11)	2.70	0.100
Good Sleep, *N (%)*	24 (68.6)	14 (77.8)	10 (58.8)		
Poor Sleep, *N (%)*	11 (31.4)	4 (22.2)	7 (41.2)		
^a^ **Duration of Sleep**				0.54	0.463
No difficulty, *N (%)*	18 (51.4)	9 (50.0)	9 (52.9)		
Little difficulty, *N (%)*	5 (14.3)	1 (5.6)	4 (23.5)		
Moderate difficulty, *N (%)*	8 (22.9)	5 (27.8)	3 (17.6)		
Severe difficulty, *N (%)*	4 (11.4)	3 (16.7)	1 (5.9)		
^a^ **Sleep Disturbance**				**6.20**	**0.013**
No difficulty, *N (%)*	3 (8.6)	1 (5.6)	2 (11.8)		
Little difficulty, *N (%)*	18 (51.4)	6 (33.3)	12 (70.6)		
Moderate difficulty, *N (%)*	13 (37.1)	10 (55.6)	3 (17.6)		
Severe difficulty, *N (%)*	1 (2.9)	1 (5.6)	0 (0.0)		
^a^ **Sleep Latency**				5.02	0.025
No difficulty, *N (%)*	5 (14.3)	2 (11.1)	3 (17.6)		
Little difficulty, *N (%)*	12 (34.3)	3 (16.7)	9 (52.9)		
Moderate difficulty, *N (%)*	13 (37.1)	9 (50.0)	4 (23.5)		
Severe difficulty, *N (%)*	5 (14.3)	4 (22.2)	1 (5.9)		
^a^ **Day Dysfunction due to Sleepiness**				0.60	0.438
No difficulty, *N (%)*	8 (22.9)	5 (27.8)	3 (17.6)		
Little difficulty, *N (%)*	20 (57.1)	7 (38.9)	13 (76.5)		
Moderate difficulty, *N (%)*	5 (14.3)	4 (22.2)	1 (5.9)		
Severe difficulty, *N (%)*	2 (5.7)	2 (11.1)	0 (0.0)		
^a^ **Sleep Efficiency**				1.81	0.179
No difficulty, *N (%)*	19 (54.3)	8 (44.4)	11 (64.7)		
Little difficulty, *N (%)*	8 (22.9)	5 (27.8)	3 (17.6)		
Moderate difficulty, *N (%)*	4 (11.4)	1 (5.6)	3 (17.6)		
Severe difficulty, *N (%)*	4 (11.4)	4 (22.2)	0 (0.0)		
^**a**^ **Overall Sleep Quality**				0.15	0.698
No difficulty, *N (%)*	5 (14.3)	2 (11.1)	3 (17.6)		
Little difficulty, *N (%)*	26 (74.3)	14 (77.8)	12 (70.6)		
Moderate difficulty, *N (%)*	3 (8.6)	1 (5.6)	2 (11.8)		
Severe difficulty, *N (%)*	1 (2.9)	1 (5.6)	0 (0.0)		
^a^ **Need Medication to Sleep**				0.32	0.574
No difficulty, *N (%)*	27 (77.1)	13 (72.2)	14 (82.4)		
Little difficulty, *N (%)*	4 (11.4)	3 (16.7)	1 (5.9)		
Moderate difficulty, *N (%)*	1 (2.9)	1 (5.6)	0 (0.0)		
Severe difficulty, *N (%)*	3 (8.6)	1 (5.6)	2 (11.8)		
^**a**^ **Perceived Cohesion Scale**	4.85 (1.14)	4.34 (1.21)	5.39 (0.77)	**7.35**	**0.007**
Belonging Factor, *Mean (SD)*	5.03 (1.06)	4.57 (1.10)	5.51 (0.78)	**7.85**	**0.005**
Morale Factor, *Mean (SD)*	4.68 (1.26)	4.11 (1.38)	5.27 (0.80)	**7.12**	**0.008**

^a^ missing 1

The infected group experienced significantly higher levels of stress (DASS-21 Stress) with χ^2^(1) = 6.74, p = 0.009, effect size: medium (ε^2^ = 0.198), as compared to the control group. More participants in the infected group experienced more severe difficulties in sleep (PSQI), χ^2^(1) = 6.20, p = 0.013, effect size: medium (ε^2^ = 0.182), as compared to the control group. The control group also reported significantly higher perception of team cohesion, χ^2^(1) = 7.35, p = 0.007, effect size: medium (ε^2^ = 0.216), as compared to the infected group. This was reflected both in belonging and morale factor, χ^2^(1) = 7.85; p = 0.005; effect size medium (ε^2^ = 0.231) for the former, and χ^2^(1) = 7.12; p = 0.008; effect size medium (ε^2^ = 0.209) for the latter.

### Psychosocial measures, perceived cohesion measure and Brief-COPE scores across time points

Descriptive information of the psychosocial measures and Brief COPE is summarised in Table 3. Across the timepoints, depressive symptoms were reported in 13.3% to 25.7%, anxiety symptoms in 13.5% to 25.7% and stress symptoms in 5.7% to 13.3% of the surveyed MHCWs. Wellbeing scores were generally moderate (66.7%-77.1%). There were 31.4% to 53.3% with poor sleep across the timepoints. Perceived cohesion scores were generally high (*M* = 4.71–5.06, *SD* = 1.14–1.99). There were no significant differences in psychosocial measures across timepoints except SWEMWBS, χ^2^(2) = 5.43, *p* = 0.07. There was a lower mean score of SWEMWBS at T2 compared to the rest of the time points.

**Table 3:** Descriptive statistics and comparison of measures and coping style across timepoints (total sample).

Measure	T1 (n = 37)	T2 (n = 35)	T3 (n = 15)	*χ* ^*2*^ */F*	p-value
**DASS-21 Stress, *Mean (SD)***	0.11 (0.31)	0.11 (0.53)	0.13 (0.35)	3.00	0.22
Normal range, *N (%)*	33 (89.2)	33 (94.3)	13 (86.7)		
Positive range, *N (%)*	4 (10.8)	2 (5.7)	2 (13.3)		
**DASS-21 Anxiety, *Mean (SD)***	0.12 (0.52)	0.46 (0.82)	0.33 (0.72)	1.14	0.57
Normal range, *N (%)*	32 (86.5)	26 (74.3)	12 (80.0)		
Positive range, *N (%)*	5 (13.5)	9 (25.7)	3 (20.0)		
**DASS-21 Depression, *Mean (SD)***	0.22 (0.58)	0.49 (0.85)	0.27 (0.70)	2.8	0.25
Normal range, *N (%)*	32 (86.5)	26 (74.3)	13 (86.7)		
Positive range, *N (%)*	5 (13.5)	9 (25.7)	2 (13.3)		
**SWEMWBS, *Mean (SD)***	24.97 (4.35)	23.87 (3.69)	24.86 (5.22)	**5.43**	**0.07**
Low, *N (%)*	4 (10.8)	5 (14.3)	2 (13.3)		
Moderate, *N (%)*	25 (67.6)	27 (77.1)	10 (66.7)		
High, *N (%)*	8 (21.6)	3 (8.6)	3 (20.0)		
**PSQI, *Mean (SD)***	6.14 (3.01)	7.06 (3.80)	6.13 (4.09)	0.39^a^	0.60
				1.64	0.44
Good Sleep, *N (%)*	23 (62.2)	24 (68.6)	7 (46.7)		
Poor Sleep, *N (%)*	14 (37.8)	11 (31.4)	8 (53.3)		
**Duration of Sleep**				1.81	0.41
No difficulty, *N (%)*	13 (35.1)	18 (51.4)	7 (46.7)		
Little difficulty, *N (%)*	16 (43.2)	5 (14.3)	3 (20.0)		
Moderate difficulty, *N (%)*	8 (21.6)	8 (22.9)	3 (20.0)		
Severe difficulty, *N (%)*	0 (0.0)	4 (11.4)	2 (13.3)		
**Sleep Disturbance**				1.73	0.42
No difficulty, *N (%)*	4 (10.8)	3 (8.6)	1 (6.7)		
Little difficulty, *N (%)*	24 (64.9)	18 (51.4)	11 (73.3)		
Moderate difficulty, *N (%)*	8 (21.6)	13 (37.1)	2 (13.3)		
Severe difficulty, *N (%)*	1 (2.7)	1 (2.9)	1 (6.7)		
**Sleep Latency**				0.35	0.84
No difficulty, *N (%)*	4 (10.8)	5 (14.3)	4 (26.7)		
Little difficulty, *N (%)*	14 (37.8)	12 (34.3)	6 (40.0)		
Moderate difficulty, *N (%)*	14 (37.8)	13 (37.1)	2 (13.3)		
Severe difficulty, *N (%)*	5 (13.5)	5 (14.3)	3 (20.0)		
**Day Dysfunction due to Sleepiness**				2.57	0.28
No difficulty, *N (%)*	13 (35.1)	8 (22.9)	5 (33.3)		
Little difficulty, *N (%)*	20 (54.1)	20 (57.1)	9 (60.0)		
Moderate difficulty, *N (%)*	3 (8.1)	5 (14.3)	1 (6.7)		
Severe difficulty, *N (%)*	1 (2.7)	2 (5.7)	0 (0.0)		
**Sleep Efficiency**				0.21	0.90
No difficulty, *N (%)*	24 (64.9)	19 (54.3)	8 (53.3)		
Little difficulty, *N (%)*	6 (16.2)	8 (22.9)	2 (13.3)		
Moderate difficulty, *N (%)*	2 (5.4)	4 (11.4)	3 (20.0)		
Severe difficulty, *N (%)*	5 (13.5)	4 (11.4)	2 (13.3)		
**Overall Sleep Quality**				0.09	0.96
No difficulty, *N (%)*	8 (21.6)	5 (14.3)	4 (26.7)		
Little difficulty, *N (%)*	26 (70.3)	26 (74.3)	10 (66.7)		
Moderate difficulty, *N (%)*	3 (8.1)	3 (8.6)	0 (0.0)		
Severe difficulty, *N (%)*	0 (0.0)	1 (2.9)	1 (6.7)		
**Need Medication to Sleep**				1.20	0.55
No difficulty, *N (%)*	32 (86.5)	27 (77.1)	13 (86.7)		
Little difficulty, *N (%)*	2 (5.4)	4 (11.4)	2 (13.3)		
Moderate difficulty, *N (%)*	2 (5.4)	1 (2.9)	0 (0.0)		
Severe difficulty, *N (%)*	1 (2.7)	3 (8.6)	0 (0.0)		
**Perceived Cohesion Scale**	5.06 (1.16)	4.85 (1.14)	4.71 (1.99)	1.15	0.32
Belonging Factor, *Mean (SD)*	5.13 (1.16)	5.03 (1.06)	4.73 (2.02)	1.19	0.31
Morale Factor, *Mean (SD)*	5.00 (1.21)	4.68 (1.26)	4.69 (1.96)	1.16	0.32
**Problem-Focused Coping, *Mean (SD)***	2.69 (0.69)	2.63 (0.71)	2.48 (0.72)	2.87	0.238
Active Coping, *Mean (SD)*	2.89 (0.94)	2.81(0.90)	2.63 (0.95)	1.27	0.531
Use of Informational Support, *Mean (SD)*	2.27 (0.97)	2.31 (0.91)	2.27 (1.00)	0.37	0.832
Positive Reframing, *Mean (SD)*	3.00 (0.72)	2.99 (0.77)	2.77 (0.70)	2.51	0.285
Planning, *Mean (SD)*	2.61 (0.83)	2.40 (0.78)	2.27 (0.68)	8.16	0.017
**Emotion-Focused Coping, *Mean (SD)***	2.24 (0.52)	2.25 (0.45)	2.14 (0.40)	0.59	0.744
Emotional Support, *Mean (SD)*	2.50 (0.90)	2.39 (1.01)	2.40 (0.74)	2.60	0.273
Venting, *Mean (SD)*	2.00 (0.75)	1.90 (0.67)	1.77 (0.62)	0.34	0.843
Humor, *Mean (SD)*	1.85 (0.80)	1.84 (0.76)	1.87 (0.67)	2.28	0.320
Acceptance, *Mean (SD)*	3.42 (0.80)	3.20 (0.77)	3.37 (0.64)	1.41	0.494
Religion, *Mean (SD)*	2.47 (1.17)	2.73 (1.13)	2.30 (1.08)	0.40	0.819
Self-blame, *Mean (SD)*	1.23 (0.43)	1.43 (0.57)	1.13 (0.30)	0.30	0.861
**Avoidant Coping, *Mean (SD)***	1.51 (0.24)	1.62 (0.34)	1.50 (0.26)	2.59	0.274
Self-distraction, *Mean (SD)*	2.68 (0.95)	2.71 (0.96)	2.63 (0.81)	3.04	0.219
Denial, *Mean (SD)*	1.20 (0.38)	1.46 (0.67)	1.13 (0.35)	3.50	0.174
Substance use, *Mean (SD)*	1.00 (0.00)	1.07 (0.25)	1.07 (0.26)	2.00	0.368
Behavioral Disengagement, *Mean (SD)*	1.15 (0.39)	1.23 (0.43)	1.17 (0.52)	2.00	0.368
**Average Score**					
Problem-focused coping	2.63	2.75	2.75		
Emotion-focused coping	2.08	2.42	2.25		
Avoidant coping	1.63	1.38	1.38		
**Normative Percentile**					
Problem-focused coping	60	67.2	67.2		
Emotion-focused coping	38	65.1	51.6		
Avoidant coping	49.1	28.2	28.2		
**Clinical Percentile**					
Problem-focused coping	47.5	55	55		
Emotion-focused coping	30	57.5	42.5		
Avoidant coping	27.5	10	10		

^a^ F-Value

Across the timepoints, surveyed MHCWs’ problem-focused coping average scores were 2.63 to 2.75, scoring higher than 60 to 67.2% of the regular individuals, and higher than 47.5% to 55% of the clinical sample. Emotion-focused coping average scores were 2.08 to 2.42, scoring higher than 38% to 65.1% of the regular individuals, and higher than 30% to 57.5% of the clinical sample. Avoidant coping average scores were 1.38 to 1.63, scoring higher than 28.2% to 49.1% of the regular individuals, and higher than 10% to 27.5% of the clinical sample. On average, it seems that the surveyed MHCWs engage in more problem-focused coping (especially at T2 and T3) and emotion-focused coping at T2, as compared to the norm data.

It was found that there were no significant changes in coping style across the timepoint. Therefore, the coping style data from time point 1 was used for subsequent analyses.

### Association between Brief-COPE and psychosocial measures

The association between T1 Brief-COPE and the psychosocial measures at T1-T2 are presented in Tables 4 and 5. The associations with PSQI are not presented as there were no significant associations (see S5 Table). At Timepoint 1, greater use of behavioural disengagement had a moderate correlation (T_b_ = 0.448, p<0.017) with higher levels of depressive symptoms (DASS-21) and a large correlation (T_b_ = 0.527, p<0.017) with higher levels of stress symptoms (DASS-21). Greater use of positive reframing was moderately correlated with higher levels of anxiety symptoms (DASS-21) (T_b_ = 0.370, p<0.017) but also moderately correlated (T_b_ = 0.349, p<0.017) with higher levels of wellbeing (SWEMWBS). Greater use of avoidant coping (T_b_ = 0.383, p<0.017) was also moderately correlated with higher levels of anxiety symptoms (DASS-21).

**Table 4:** Correlation between Time 1 Brief-COPE with Timepoint 1 (n = 37) & 2 (n = 35) psychosocial variables- DASS-21 & SWEMWBS.

	1	2	3	4	5
***Timepoint 1***					
**Problem-Focused Coping**	0.046	0.221	-0.045	0.126	0.114
Active Coping	0.016	0.182	-0.077	0.021	0.116
Use of Informational Support	0.210	0.144	0.143	0.109	0.034
Positive Reframing	0.083	0.370*	-0.019	0.349*	0.027
Planning	-0.099	0.125	-0.144	0.030	0.141
**Emotion-Focused Coping**	0.174	0.330	0.168	0.039	0.136
Emotional Support	0.204	0.281	0.277	0.057	0.163
Venting	0.157	0.297	0.041	0.042	0.048
Humor	0.276	0.191	0.290	-0.050	0.068
Acceptance	-0.021	0.233	0.016	0.180	0.151
Religion	0.101	0.303	0.077	-0.069	0.000
Self-blame	0.081	-0.089	0.120	-0.076	0.032
**Avoidant Coping**	0.237	0.383*	0.211	0.087	-0.028
Self-distraction	0.113	0.311	0.065	0.179	-0.046
Denial	0.045	0.113	-0.035	-0.197	0.082
Substance use	-	-	-	-	-
Behavioral Disengagement	0.448*	0.011	0.527*	-0.199	-0.155
***Timepoint 2***					
**Problem-Focused Coping**	0.105	-0.013	0.000	0.253	-0.012
Active Coping	0.072	-0.006	-0.141	0.226	0.032
Use of Informational Support	0.058	0.043	0.219	0.239	-0.080
Positive Reframing	-0.012	-0.190	0.083	0.211	0.073
Planning	0.144	0.057	-0.076	0.294	-0.069
**Emotion-Focused Coping**	0.105	0.026	-0.015	0.348*	-0.114
Emotional Support	-0.050	-0.026	0.072	0.385*	-0.087
Venting	0.160	0.026	0.022	0.278	-0.178
Humor	0.110	0.165	-0.084	0.271	-0.132
Acceptance	0.035	-0.078	-0.185	0.309	0.058
Religion	0.049	-0.088	0.011	-0.062	-0.051
Self-blame	-0.126	0.017	0.159	0.223	0.000
**Avoidant Coping**	0.255	0.163	0.038	0.282	-0.072
Self-distraction	0.276	0.135	0.146	0.256	-0.153
Denial	0.058	-0.088	-0.147	-0.142	0.143
Substance use	-	-	-	-	-
Behavioral Disengagement	-0.006	0.218	-0.086	0.046	0.068

1: DASS-21 Depression; 2: DASS-21 Anxiety; 3: DASS-21 Stress; 4: SWEMWBS level of wellbeing, 5: Perceived Cohesion Scale. Reported correlation is significant at the *p<0.017 level.

**Table 5:** Correlation between Time 1 Brief-COPE with Timepoint 2 (n = 35) psychosocial variables- PSQI.

	1	2	3	4	5	6	7	8
***Timepoint 2***								
**Problem-Focused Coping**	-0.356*	-0.035	-0.078	-0.157	-0.113	-0.274	-0.016	-0.227
Active Coping	-0.261	-0.096	-0.122	-0.179	-0.130	-0.197	-0.105	-0.240
Use of Informational Support	-0.456*	0.102	-0.127	-0.068	-0.167	-0.235	-0.012	-0.243
Positive Reframing	-0.279	-0.019	0.007	-0.163	0.042	-0.398*	-0.015	-0.144
Planning	-0.231	-0.094	0.011	-0.163	-0.023	-0.209	0.082	-0.163
**Emotion-Focused Coping**	-0.244	0.077	0.116	-0.044	-0.038	-0.133	0.155	-0.079
Emotional Support	-0.291	0.057	0.092	0.005	-0.127	-0.136	0.077	-0.108
Venting	-0.252	0.127	0.066	0.104	0.025	-0.076	0.083	-0.040
Humor	-0.076	0.227	0.076	0.051	-0.125	0.034	0.138	0.000
Acceptance	-0.042	-0.160	0.009	-0.261	0.177	-0.244	0.000	-0.083
Religion	-0.235	-0.101	0.131	-0.140	-0.011	-0.136	0.114	-0.064
Self-blame	-0.241	0.089	-0.056	0.218	-0.316	0.142	0.051	-0.121
**Avoidant Coping**	-0.089	0.172	0.096	0.203	-0.014	-0.209	0.213	0.039
Self-distraction	-0.044	0.165	-0.006	0.162	-0.009	-0.372*	0.095	-0.034
Denial	-0.090	-0.101	0.078	-0.003	0.021	0.030	0.024	0.008
Substance use	-	-	-	-	-	-	-	-
Behavioral Disengagement	-0.190	0.232	0.008	0.267	-0.180	0.384	0.235	0.131

1: Duration of Sleep; 2: Sleep Disturbance; 3: Sleep Latency; 4: Day Dysfunction due to Sleepiness; 5: Sleep Efficiency; 6: Overall Sleep Quality; 7: Need Medication to Sleep; 8: PSQI Total. Reported correlation is significant at the *p<0.017 level.

At T2, greater use of emotion-focused coping was moderately correlated (T_b_ = 0.348, p<0.017) with higher levels of wellbeing (SWEMWBS), particularly greater use of emotional support (T_b_ = 0.385, p<0.017; moderate).

T1 Brief-COPE was not significantly correlated with T2 PSQI total score. However, when broken down into its subscales, greater use of problem-focused coping was moderately correlated (T_b_ = -0.356, p<0.017) with shorter duration of sleep, specifically greater use of informational support (T_b_ = -0.456, p<0.017; moderate). Greater use of positive reframing (T_b_ = -0.398, p<0.017) and self-distraction ((T_b_ = -0.372, p<0.017) were moderately correlated with worse overall sleep quality.

The association between T1 Brief-COPE and T3 psychosocial measures are not presented as there were no significant associations (see S6 & S7 Tables).

### Thematic analysis

Only 17 participants (infected group- n = 13, control group- n = 4) agreed to participate in the in-depth interview.

*Q1*: *Thinking back to the time when COVID-19 was first announced in Wuhan China*, *what were some of the thoughts and feelings going through you*?

Most participants reported feeling worried about being infected or infecting others at home. They also reported feeling scared as information about COVID-19 was limited and vaccinations did not have sufficient data to allay fears related to side effects. Others felt helpless, especially during the incubation period, as they were aware that they could have been exposed but received no confirmation, and no reliable vaccination at that point in time, as well as difficulties managing patients who did not comply with infection control measures (e.g., wearing face masks) and the travel restrictions (especially for foreigners).

*Q2*: *How did you and your team react when you were informed that there was a patient diagnosed with COVID-19 in the ward that you are working in*?

Some participants were in disbelief and shock, and most of the staff felt scared and worried as they were concerned about infection or infecting others whom they come to contact with at home, especially children and elderly parents. Others felt confused over the rapidly changing infection control protocols. Nevertheless, some reported feeling confident of the work processes as they adjusted to the new guidelines and reassured as they observed consistent teamwork and support within ward team.

*Q3*: *How did you and/or your team cope with the news*?

Participants generally coped through seeking instrumental support. For instance, MHCWs immediately complied and donned on personal protective equipment and familiarised themselves with the new infection control guidelines. They also reached out to their immediate supervisors for guidance, clarifications, and feedback to ensure that patient care is not disrupted.

Emotional support was also commonly reported, with their supervisors constantly checking in on their wellbeing, encouraging the team to ‘think positively’ and to exercise self-care. The availability of the supervisors for guidance, their flexibility and responses to feedback, and emotional support made most of the participants feel reassured and supported during these difficult times.

*Q4*: *Looking back*, *what have helped to alleviate the impact of the confirmed case of COVID-19 in the ward*?

Responses were categorised into three main themes–self, team, and hospital management. Themes and examples are presented in Table 6.

**Table 6:** Q4 What have helped to alleviate the impact of the confirmed case of COVID-19 in the ward?

Category	Theme	Subtheme	Illustrative Example
Self	Distraction		*Do something that I can redirect or refocus my attention after work like exercise*
	Social support		*Speak to my family members*, *some of my friends also*. *Talk about the situation*, *chit chat*, *feel better*
	Active Coping	Health oriented behaviours	*Take vitamins*
			*Actually I am more of a home person so just stay at home*. *Ok la*, *I think take care of the hygiene is sufficient*.
		Alternatives	*we can’t go out and spend time as we used to but like my friends and I will try to have a video chat*
	Religion		*I just like pray more*.
			
Team	Existing Team Support	Teamwork	*So during that period*, *we are going strong*, *no one is taking MC (medical certificate given after consultation with doctor) or being sick*. *And also none of us took leave at that point of time*
			*Make sure we don the PPE (personal protective equipment) correctly and then we support each other*.
		Social support	*I mean my NC (Nurse Clinician- supervisor/manager) quite supportive … they will sit down asking like any issue*, *any concern your staff want to address… to help to solve the problem*.
	Rewards and recognition	Appreciation	*The medical team provided us some food*, *drinks*. *They appreciate our help to look after their patients*.
	Active coping	Acceptance	*… when we heard the confirmed cases in our institution*, *we accept the fact that we may have it sooner or later also so the staff they feel worried*, *and they feel scared*
		Compliance with instructions	*talk to management- they will tell us what to do*, *how to do*
			
Management	Prompt responses to pandemics		*very prompt change in the protocol*, *change in the management*, *in that the management has reinforced*, *like you know*, *immediately PPE*, *immediately swab everything and we have to segregate this and that*.
			*… allow us to do the COVID test*, *the staff to reassure that the result to make us more comfortable*. *Otherwise*, *they also provide the accommodation*, *like those that stay with family they may worry*, *… and also provide some food … air cooler as we wearing PPE very hot*
	Genuinely checking in on ground needs and wellbeing	Openness to feedback and ground needs	*(hospital leadership)*, *actually did a zoom with us … it was not just a one time zoom*, *it was quite a few times*.
		Responsiveness to feedback and ground needs	*Whatever we request*, *the management accede*, *mobile air-conditioning*, *own tee shirt under PPE etc*.
	Leadership presence on the ground		*It’s good to see (hospital leadership) on the ground*, *(even on Public Holidays when things happened*, *turning ward into isolation ward)*, *that our efforts are seen*

Participants mainly utilised distraction techniques to alleviate the stress imposed by the confirmed case of COVID-19 in the ward. They gravitated towards healthy distractions such as exercising and developing hobbies such as gardening and crocheting. Most switched to video calls to stay connected with their family members and friends who are overseas, while others turned to health-oriented behaviours such as taking vitamins, minimise going out or reading to understand more about COVID-19. Some turned to religion and prayed more or practiced mindfulness.

On a team level, participants mainly reported accepting the news of the case and actively coping with rapidly changing infection control measures. The existing team support was also put to good use, as participants felt supported in the current team. This camaraderie was further strengthened when it was observed that peers tried to minimise absenteeism to avoid placing additional manpower strains to the team and looked out for one another. The appreciation from members of the multidisciplinary team was also felt through food items delivered to cheer nursing staff on.

Finally, the presence of hospital leadership was highly felt by the MHCWs. Participants highlighted the genuine care and concern from hospital leadership such as the multiple video conferencing sessions to check in on their welfare and seeking their feedback. Their confidence in the leadership was further strengthened when they witnessed senior management being actively involved and helping out on the ground and by their receptiveness and efficiency with which the hospital leadership responded to their feedback to improve working conditions (e.g., mobile air conditioning) and address their worries of infecting family members (e.g., alternative accommodation).

*Q5*: *Do you have any thoughts on the areas for improvement in the hospital’s response in managing the COVID-19 cases*, *as well as the response and support for staff working in the affected wards*?

There are seven main areas which participants reported that the psychiatric institute had done well in their responses to COVID-19 (See S8 Table). Most MHCWs perceived that the institute had been prompt in containing the infection, followed by clear and prompt updates from hospital leadership and within their teams. Other factors include efficiency in hospital and team management’s response to feedback and requests, cohesiveness within teams.

Participants highlighted several areas for improvement (See S8 Table). In terms of communication, participants hoped that visitors can be better managed. Though rapidly changing infection control guidelines confused them, this was overcome in some teams through good group communications and handovers. It was proposed that management could prepare ahead of time when there are early warning signs for future pandemics so that they can have better mental preparation.

Other helpful suggestions include enhancing work environment such as providing a cooling work environment and more rest areas to allow more restful breaks. Staff also hope to maintain isolation wards, which can be converted at short notice to house infected patients.

## Discussion

The current study examined the psychosocial impact of COVID-19 in a mental health setting, as well as the relationship between coping strategies and psychological wellbeing during a pandemic. This study also used an in-depth interview to understand the role of organisational interventions in moderating pandemic stress. The quantitative results were partly consistent with our hypothesis of MHCWs from infected wards experiencing greater psychological distress than the non-infected wards. This was seen at T2 where there were more staff from infected wards reporting significantly higher stress levels and more sleep disturbances, as compared to the non-infected wards. The results also provided partial support for our second hypothesis that positive coping styles are likely to buffer psychological distress as not all positive coping styles had significant positive correlations with well-being. For instance, problem-focused coping style (positive coping style) had significant correlations with both positive and negative outcomes. Team cohesion did not have significant correlations with the psychological outcomes.

Using non-infected wards as the control, the results demonstrated that MHCWs experienced higher stress and sleep disturbance during a pandemic. Although MHCWs from infected wards were more stressed at one point, a result consistent with another study [51], MHCWs from non-infected wards also reported similar levels of distress as those from infected wards for the other time points. This seems to echo Tan et al. [25]’s report that exposure in terms of proximity with the virus does not negate the internal psychological distress experienced by HCWs. Rather, they found that non-medical HCWs experienced higher anxiety than medical HCWs. This suggests that all groups of HCWs and MHCWs are negatively impacted by the pandemic but may require different interventions due to differing needs.

While it is clear avoidant coping style would be correlated with poorer psychological wellbeing, the evidence for relationship between emotion-focused coping style and wellbeing has been mixed [52]. Our results, however, supports that emotion-focused coping (specifically emotional support) has a protective effect (i.e., higher well-being), a finding consistent with Eisenbeck et al.’s [53] analysis of data from 30 countries reporting adaptive coping, including emotional and instrumental support, was related to better wellbeing and fewer depressive symptoms. On the other hand, our finding for problem-focused coping is in contrast with previous literature that found it to be protective against psychological symptoms [54]. Although it is unclear if MHCWs with higher anxiety engage in more positive reframing or vice versa, it is possible that sleep quality and quantity were affected when MHCWs engaged in more informational support and positive reframing, as more time, cognitive and behavioural effort is needed [55].

Consistent with previous literature, our in-depth interviews have highlighted the importance of institutional measures as a key resource that supports, sustains and offsets demands [8, 56, 57] of HCWs during pandemics, thereby mitigating burnout [58]. Like the needs mentioned in Poh et al.’s [24] study, our participants also expressed concerns over emotional security, workplace safety, confidence, and trust in leadership. While Poh et al. [24] called for organisational interventions to address their participants’ requests for effective and timely communications of pandemic information, expression of encouragement and gratitude to HCWs, regular engagement sessions between leaders and the staff to realign perspectives, our participants mainly reported gratitude towards team and organisation leadership for prompt actions and engagement. Specifically, they appreciated the responsiveness of the institutional support, such as in acting on their request for air conditioners, providing meals and refreshments as a form of tangible encouragement for their hard work. They were also thankful for the emotional and information support from nursing leader, including frequent check-ins, encouragement to ‘think positively’ and self-care reminders, as well as availing themselves to clarify infection control protocols and guidelines. Further, the presence of team and hospital leaders on the ground, built trust and confidence in the leadership. As highlighted by the participants, ‘*It’s good to see (senior management) on the ground*, *that our efforts are seen’*. This may have impressed upon the MHCWs that hospital leadership shared their pain, and validated their contributions and sacrifices, hence fostering a sense of teamwork and belonging [59].

### Study limitations

We had used recall method for the first time point survey due to the time lapse by the time the study was conducted. We also had considerable dropouts by the third timepoint. The sample size is small, but this was dealt with using effect size. The study was also strengthened with qualitative information from in-depth interviews. Finally, correlational relationships are unable to provide causal explanations.

### Conclusion

MHCWs face considerable stress and sleep disturbance during COVID-19, especially MHCWs working in infected wards. However, MHCWs in non-infected wards also faced some level of distress. Institutional leadership and interventions mitigate the pandemic burden on MHCWs and can enhance individual’s emotional coping. These findings may contribute to improvements in future pandemic management.

## Supporting information

S1 Table Descriptive statistics of measures- comparison between drop-out and non-drop-out Group between (Visit 2 and 3). (DOCX)

S2 Table Comparison of study measures between drop-out group and non-drop out group (Visit 2 and 3). (DOCX)

S3 Table Descriptive statistics of measures- comparison between target and control group for Visit 1. (DOCX)

S4 Table Descriptive statistics of measures- comparison between target and control group for Visit 3. (DOCX)

S5 Table Correlation between time 1 Brief-COPE with time point 1 psychosocial variable- PSQI (n = 37). (DOCX)

S6 Table Correlation between time 1 Brief-COPE with time point 3 psychosocial variables- DASS-21 & SWEMWBS (n = 15). (DOCX)

S7 Table Correlation between time 1 Brief-COPE with time point 3 psychosocial variable- PSQI (n = 15). (DOCX)

S8 Table Q5 Do you have any thoughts on the areas for improvement in IMH’s response in managing the COVID-19 cases. (DOCX)
