# Supplementary material for: Psychological well-being of healthcare workers during COVID-19 in a mental health institution
Source: PLoS One. 2024 Mar 18;19(3):e0300329. doi: 10.1371/journal.pone.0300329 (PMC10947715; doi:10.1371/journal.pone.0300329)
Supplement: S1 Table — (DOCX) [file pone.0300329.s001.docx]

**Supporting Information**

**Table 1**

Descriptive Statistics of Measures- comparison between Drop-out and Non-drop-out Group (Visit 2 and 3)

| Characteristic | Drop-out group (n=20) | Non-drop-out group (n=15) | *X*^2^ | p-value |
| --- | --- | --- | --- | --- |
| **Gender, *N (%)*** |  |  | 3.34 | 0.068 |
| Male | 13 (65.0) | 5 (33.3) |  |  |
| Female | 7 (35.0) | 10 (66.7) |  |  |
| **Age range, N (%)** |  |  | 0.29 | 0.589 |
| 20-30 | 7 (35.0) | 2 (13.3) |  |  |
| 31-40 | 6 (30.0) | 8 (53.3) |  |  |
| 41-50 | 4 (20.0) | 2 (13.3) |  |  |
| 51-60 | 3 (15.0) | 3 (20.0) |  |  |
| **Ethnicity, *N (%)*** |  |  | 0.92 | 0.336 |
| Chinese | 6 (30.0) | 7 (46.7) |  |  |
| Malay | 4 (20.0) | 3 (20.0) |  |  |
| Indian | 3 (15.0) | 1 (6.7) |  |  |
| Others | 7 (35.0) | 4 (26.7) |  |  |
| **Marital status, *N (%)*** |  |  | 0.16 | 0.691 |
| Single | 8 (40.0) | 5 (33.3) |  |  |
| Married/living with  someone as if  married | 12 (60.0) | 10 (66.7) |  |  |
| **Children, N (%)** |  |  | 0.34 | 0.562 |
| Yes | 10 (50.0) | 9 (60.0) |  |  |
| No | 10 (50.0) | 6 (40.0) |  |  |
|  |  |  |  |  |
| **Years in Service, N (%)** |  |  | 0.39 | 0.535 |
| 0-5 years | 7 (35.0) | 5 (33.3) |  |  |
| - 1. years | 9 (45.0) | 6 (40.0) |  |  |
| >15 years | 4 (20.0) | 4 (26.7) |  |  |
|  |  |  |  |  |
| **Job Designation, N (%)** |  |  | 2.40 | 0.122 |
| Managerial Nurse | 4 (20.0) | 6 (40.0) |  |  |
| Staff Nurse | 10 (50.0) | 7 (46.7) |  |  |
| Assistant Nurse | 4 (20.0) | 2 (13.3) |  |  |
| Psychiatrist | 1 (5.0) | 0 (0) |  |  |
| Peer Support  Specialist | 1 (5.0) | 0 (0) |  |  |
| **Chronic Physical Condition, N (%)** |  |  | 0.02 | 0.891 |
| Yes | 3 (15.0) | 2 (13.3) |  |  |
| No | 17 (85.0) | 13 (86.7) |  |  |
