# Supplementary material for: Psychological well-being of healthcare workers during COVID-19 in a mental health institution
Source: PLoS One. 2024 Mar 18;19(3):e0300329. doi: 10.1371/journal.pone.0300329 (PMC10947715; doi:10.1371/journal.pone.0300329)
Supplement: S2 Table — (DOCX) [file pone.0300329.s002.docx]

**Supporting Information**

**Table 2**

*Comparison of Study Measures between drop-out group and non-drop-out group (Visit 2 and 3)*

| Measure | Drop-out  group  (n=20) | Non-drop-out group  (n=15) | *X*^2^ | p-value |
| --- | --- | --- | --- | --- |
|  |  |  |  |  |
| **DASS-21 Stress, *Mean (SD)*** | 8.60 (7.05) | 6.13 (5.21) | 0.87 | 0.347 |
| Normal range, *N (%)* | 19 (95.0) | 14 (70.0)  1 (30.0) |  |  |
| Positive range, *N (%)* | 1 (5.0) |  |  |  |
| **DASS-21 Anxiety, *Mean (SD)*** | 4.90 (4.83) | 3.87 (4.50) | 0.44 | 0.506 |
| Normal range, *N (%)* | 14 (70.0) | 12 (80.0) |  |  |
| Positive range, *N (%)* | 6 (30.0) | 3 (20.0) |  |  |
| **DASS-21 Depression, *Mean (SD)*** | 6.40 (5.86) | 5.07 (5.12) | 0.44 | 0.505 |
| Normal range, *N (%)* | 14 (70.0) | 12 (80.0) |  |  |
| Positive range, *N (%)* | 6 (30.0) | 3 (20.0) |  |  |
| **SWEMWBS, *Mean (SD)*** | 23.64 (4.44) | 24.18 (2.49) | 0.36 | 0.546 |
| Low, *N (%)* | 4 (20.0) | 1 (6.7) |  |  |
| Moderate, *N (%)* | 13 (65.0) | 14 (93.3) |  |  |
| High, *N (%)* | 3 (15.0) | 0 (0.0) |  |  |
| **^a^PSQI, *Mean (SD)*** | 7.30 (4.22) | 6.73 (3.28) | 0.00 | 0.972 |
|  |  |  |  |  |
| Good Sleep, *N (%)* | 9 (45.0) | 7 (46.7) |  |  |
| Poor Sleep, *N (%)* | 10 (50.0) | 8 (53.3) |  |  |
| ^a^**Duration of Sleep** |  |  | 0.52 | 0.472 |
| No difficulty, *N (%)* | 11 (55.0) | 7 (46.7) |  |  |
| Little difficulty, *N (%)* | 3 (15.0) | 2 (13.3) |  |  |
| Moderate difficulty, *N (%)* | 4 (20.0) | 4 (26.7) |  |  |
| Severe difficulty, *N (%)* | 2 (10.0) | 2 (13.3) |  |  |
| ^a^**Sleep Disturbance** |  |  | 0.97 **6.20** | 0.325 |
| No difficulty, *N (%)* | 2 (10.0) | 1 (6.7) |  |  |
| Little difficulty, *N (%)* | 8 (40.0) | 10 (66.7) |  |  |
| Moderate difficulty, *N (%)* | 9 (45.0) | 4 (26.7) |  |  |
| Severe difficulty, *N (%)* | 1 (5.0) | 0 (0.0) |  |  |
| ^a^**Sleep Latency** |  |  | 0.00 | 0.956 |
| No difficulty, *N (%)* | 3 (15.0) | 2 (13.3) |  |  |
| Little difficulty, *N (%)* | 7 (35.0) | 5 (33.3) |  |  |
| Moderate difficulty, *N (%)* | 6 (30.0) | 7 (46.7) |  |  |
| Severe difficulty, *N (%)* | 4 (20.0) | 1 (6.7) |  |  |
| ^a^**Day Dysfunction due to Sleepiness** |  |  | 0.01 | 0.937 |
| No difficulty, *N (%)* | 5 (25.0) | 3 (20.0) |  |  |
| Little difficulty, *N (%)* | 10 (50.0) | 10 (66.7) |  |  |
| Moderate difficulty, *N (%)* | 3 (15.0) | 2 (13.3) |  |  |
| Severe difficulty, *N (%)* | 2 (10.0) | 0 (0.0) |  |  |
| ^a^**Sleep Efficiency** |  |  | 0.29 | 0.590 |
| No difficulty, *N (%)* | 11 (55.0) | 8 (53.3) |  |  |
| Little difficulty, *N (%)* | 6 (30.0) | 2 (13.3) |  |  |
| Moderate difficulty, *N (%)* | 1 (5.0) | 3 (20.0) |  |  |
| Severe difficulty, *N (%)* | 2 (10.0) | 2 (13.3) |  |  |
| **^a^Overall Sleep Quality** |  |  | 2.17 | 0.141 |
| No difficulty, *N (%)* | 2 (10.0) | 3 (20.0) |  |  |
| Little difficulty, *N (%)* | 14 (70.0) | 12 (80.0) |  |  |
| Moderate difficulty, *N (%)* | 3 (15.0) | 0 (0.0) |  |  |
| Severe difficulty, *N (%)* | 1 (5.0) | 0 (0.0) |  |  |
| ^a^**Need Medication to Sleep** |  |  | 0.02 | 0.902 |
| No difficulty, *N (%)* | 15 (75.0) | 12 (80.0) |  |  |
| Little difficulty, *N (%)* | 2 (10.0) | 2 (13.3) |  |  |
| Moderate difficulty, *N (%)* | 1 (5.0) | 0 (0.0) |  |  |
| Severe difficulty, *N (%)* | 2 (10.0) | 1 (6.7) |  |  |
| **^a^Perceived Cohesion Scale** | 4.59 (1.22) | 5.20 (0.95) | 2.39 | 0.122 |
| Belonging Factor, *Mean (SD)* | 4.80 (1.11) | 5.33 (0.93) | 2.53 | 0.112 |
| Morale Factor, *Mean (SD)* | 4.38 (1.38) | 5.07 (1.00) | 2.20 | 0.138 |
| **Problem-Focused Coping, *Mean (SD)*** | 2.72 (0.69) | 2.51 (0.75) | 0.62 (0.72) | 0.432 |
|  |  |  |  |  |
| **Emotion-Focused Coping, *Mean (SD)*** | 2.33 (0.50) | 2.13 (0.37) | 0.94 (0.40) | 0.332 |
| **Avoidant Coping, *Mean (SD)*** | 1.76 (0.36) | 1.43 (0.22) | 7.74 (0.26) | 0.005* |
| Self-distraction | 2.93 (0.98) | 2.43 (0.88) | 1.66 | 0.197 |
| Denial | 1.68 (0.77) | 1.17 (0.36) | 5.78 | 0.016* |
| Substance use | 1.10 (0.31) | 1.03 (0.13) | 0.17 | 0.680 |
| Behavioral Disengagement  Disengagement | 1.33 (0.49) | 1.10 (0.28) | 2.25 | 0.133 |
|  |  |  |  |  |
|  |  |  |  |  |
|  |  |  |  |  |

^a^ missing 1; * significant at 0.017 level
