# Supplementary material for: Psychological well-being of healthcare workers during COVID-19 in a mental health institution
Source: PLoS One. 2024 Mar 18;19(3):e0300329. doi: 10.1371/journal.pone.0300329 (PMC10947715; doi:10.1371/journal.pone.0300329)
Supplement: S3 Table — (DOCX) [file pone.0300329.s003.docx]

**Supporting Information**

**Table 3**

Descriptive Statistics of Measures- comparison between Target and Control Group for Visit 1

| Measure | Total (n=37) | Target group (n=18) | Control group (n=19) | *X*^2^ | p-value |
| --- | --- | --- | --- | --- | --- |
|  |  |  |  |  |  |
| **DASS-21 Stress, *Mean (SD)*** | 0.11 (0.31) | 7.33 (6.06) | 4.84 (5.18) | 1.67 | 0.196 |
| Normal range, *N (%)* | 33 (89.2) | 15 (83.3) | 18 (94.7)  1 (5.3) |  |  |
| Positive range, *N (%)* | 4 (10.8) | 3 (16.7) |  |  |  |
| **DASS-21 Anxiety, *Mean (SD)*** | 0.12 (0.52) | 4.00 (3.50) | 2.74 (2.60) | 1.16 | 0.282 |
| Normal range, *N (%)* | 32 (86.5) | 15 (83.3) | 17 (89.5) |  |  |
| Positive range, *N (%)* | 5 (13.5) | 3 (16.7) | 2 (10.5) |  |  |
| **DASS-21 Depression, *Mean (SD)*** | 0.22 (0.58) | 4.11 (5.06) | 3.68 (3.90) | 0.04 | 0.837 |
| Normal range, *N (%)* | 32 (86.5) | 14 (77.8) | 18 (94.7) |  |  |
| Positive range, *N (%)* | 5 (13.5) | 4 (22.2) | 1 (5.3) |  |  |
| **SWEMWBS, *Mean (SD)*** | 24.97 (4.35) | 25.08 (4.12) | 24.87 (4.67) | 0.11 | 0.736 |
| Low, *N (%)* | 4 (10.8) | 2 (11.1) | 2 (10.5) |  |  |
| Moderate, *N (%)* | 25 (67.6) | 12 (66.7) | 13 (68.4) |  |  |
| High, *N (%)* | 8 (21.6) | 4 (22.2) | 4 (21.1) |  |  |
| **PSQI, *Mean (SD)*** | 6.14 (3.01) | 6.94 (3.26) | 5.37 (2.61) | 3.28 | 0.070 |
|  |  |  |  |  |  |
| Good Sleep, *N (%)* | 23 (62.2) | 13 (72.2) | 10 (52.6) |  |  |
| Poor Sleep, *N (%)* | 14 (37.8) | 5 (27.8) | 9 (47.4) |  |  |
| **Duration of Sleep** |  |  |  | 0.96 | 0.327 |
| No difficulty, *N (%)* | 13 (35.1) | 6 (33.3) | 7 (36.8) |  |  |
| Little difficulty, *N (%)* | 16 (43.2) | 6 (33.3) | 10 (52.6) |  |  |
| Moderate difficulty, *N (%)* | 8 (21.6) | 6 (33.3) | 2 (10.5) |  |  |
| Severe difficulty, *N (%)* | 0 (0.0) | 0 (0.0) | 0 (0.0) |  |  |
| **Sleep Disturbance** |  |  |  | 2.22 | 0.136 |
| No difficulty, *N (%)* | 4 (10.8) | 1 (5.6) | 3 (15.8) |  |  |
| Little difficulty, *N (%)* | 24 (64.9) | 11 (61.1) | 13 (68.4) |  |  |
| Moderate difficulty, *N (%)* | 8 (21.6) | 5 (27.8) | 3 (15.8) |  |  |
| Severe difficulty, *N (%)* | 1 (2.7) | 1 (5.6) | 0 (0.0) |  |  |
| **Sleep Latency** |  |  |  | 1.97 | 0.161 |
| No difficulty, *N (%)* | 4 (10.8) | 2 (11.1) | 2 (10.5) |  |  |
| Little difficulty, *N (%)* | 14 (37.8) | 4 (22.2) | 10 (52.6) |  |  |
| Moderate difficulty, *N (%)* | 14 (37.8) | 9 (50.0) | 5 (26.3) |  |  |
| Severe difficulty, *N (%)* | 5 (13.5) | 3 (16.7) | 2 (10.5) |  |  |
| **Day Dysfunction due to Sleepiness** |  |  |  | 0.005 | 0.946 |
| No difficulty, *N (%)* | 13 (35.1) | 7 (38.9) | 6 (31.6) |  |  |
| Little difficulty, *N (%)* | 20 (54.1) | 8 (44.4) | 12 (63.2) |  |  |
| Moderate difficulty, *N (%)* | 3 (8.1) | 2 (11.1) | 1 (5.3) |  |  |
| Severe difficulty, *N (%)* | 1 (2.7) | 1 (5.6) | 0 (0.0) |  |  |
| **Sleep Efficiency** |  |  |  | 0.269 | 0.604 |
| No difficulty, *N (%)* | 24 (64.9) | 11 (61.1) | 13 (68.4) |  |  |
| Little difficulty, *N (%)* | 6 (16.2) | 3 (16.7) | 3 (15.8) |  |  |
| Moderate difficulty, *N (%)* | 2 (5.4) | 1 (5.6) | 1 (5.3) |  |  |
| Severe difficulty, *N (%)* | 5 (13.5) | 3 (16.7) | 2 (10.5) |  |  |
| **Overall Sleep Quality** |  |  |  | 5.05 | 0.025 |
| No difficulty, *N (%)* | 8 (21.6) | 2 (11.1) | 6 (31.6) |  |  |
| Little difficulty, *N (%)* | 25 (67.6) | 12 (66.7) | 13 (68.4) |  |  |
| Moderate difficulty, *N (%)* | 4 (10.8) | 4 (22.2) | 0 (0.0) |  |  |
| Severe difficulty, *N (%)* | 0 (0.0) | 0 (0.0) | 0 (0.0) |  |  |
| **Need Medication to Sleep** |  |  |  | 0.26 | 0.609 |
| No difficulty, *N (%)* | 32 (86.5) | 15 (83.3) | 17 (89.5) |  |  |
| Little difficulty, *N (%)* | 2 (5.4) | 1 (5.6) | 1 (5.3) |  |  |
| Moderate difficulty, *N (%)* | 2 (5.4) | 2 (11.1) | 1 (5.3) |  |  |
| Severe difficulty, *N (%)* | 1 (2.7) | 0 (0.0) | 0 (0.0) |  |  |
| **Perceived Cohesion Scale** | 5.06 (1.16) | 4.75 (1.38) | 5.36 (0.83) | 1.77 | 0.183 |
| Belonging Factor, *Mean (SD)* | 5.13 (1.16) | 4.85 (1.38) | 5.39 (0.86) | 1.45 | 0.229 |
| Morale Factor, *Mean (SD)* | 5.00 (1.21) | 4.65 (1.47) | 5.33 (0.82) | 1.82 | 0.178 |
|  |  |  |  |  |  |
|  |  |  |  |  |  |
