# Supplementary material for: Psychological well-being of healthcare workers during COVID-19 in a mental health institution
Source: PLoS One. 2024 Mar 18;19(3):e0300329. doi: 10.1371/journal.pone.0300329 (PMC10947715; doi:10.1371/journal.pone.0300329)
Supplement: S4 Table — (DOCX) [file pone.0300329.s004.docx]

**Supporting Information**

**Table 4**

Descriptive Statistics of Measures- comparison between Target and Control Group for Visit 3

| Measure | Total (n=15) | Target group (n=5) | Control group (n=10) | *X*^2^ | p-value |
| --- | --- | --- | --- | --- | --- |
|  |  |  |  |  |  |
| **DASS-21 Stress, *Mean (SD)*** | 4.40 (5.46) | 5.60 (7.13) | 3.80 (4.76) | 0.20 | 0.658 |
| Normal range, *N (%)* | 13 (86.7) | 4 (80.0) | 9 (90.0)  1 (10.0) |  |  |
| Positive range, *N (%)* | 2 (13.3) | 1 (20.0) |  |  |  |
| **DASS-21 Anxiety, *Mean (SD)*** | 3.33 (3.44) | 4.00 (3.74) | 3.00 (3.43) | 0.576 | 0.448 |
| Normal range, *N (%)* | 32 (86.5) | 4 (80.0) | 8 (80.0) |  |  |
| Positive range, *N (%)* | 5 (13.5) | 1 (20.0) | 2 (20.0) |  |  |
| **DASS-21 Depression, *Mean (SD)*** | 3.60 (4.79) | 4.40 (5.73) | 3.20 (4.54) | 0.20 | 0.655 |
| Normal range, *N (%)* | 32 (86.5) | 4 (80.0) | 9 (90.0) |  |  |
| Positive range, *N (%)* | 5 (13.5) | 1 (20.0) | 1 (10.0) |  |  |
| **SWEMWBS, *Mean (SD)*** | 24.86 (5.22) | 24.25 (6.14) | 25.17 (5.03) | 0.55 | 0.460 |
| Low, *N (%)* | 2 (13.3) | 1 (20.0) | 1 (10.0) |  |  |
| Moderate, *N (%)* | 10 (66.7) | 3 (60.0) | 7 (70.0) |  |  |
| High, *N (%)* | 3 (20.0) | 1 (20.0) | 2 (20.0) |  |  |
| **PSQI, *Mean (SD)*** | 6.13 (4.09) | 8.00 (5.75) | 5.20 (2.90) | 1.11 | 0.293 |
|  |  |  |  |  |  |
| Good Sleep, *N (%)* | 7 (46.7) | 1 (20.0) | 6 (60.0) |  |  |
| Poor Sleep, *N (%)* | 8 (53.3) | 4 (80.0) | 4 (40.0) |  |  |
| **Duration of Sleep** |  |  |  | 1.23 | 0.268 |
| No difficulty, *N (%)* | 7 (46.7) | 1 (20.0) | 6 (60.0) |  |  |
| Little difficulty, *N (%)* | 3 (20.0) | 2 (40.0) | 1 (10.0) |  |  |
| Moderate difficulty, *N (%)* | 3 (20.0) | 1 (20.0) | 2 (20.0) |  |  |
| Severe difficulty, *N (%)* | 2 (13.3) | 1 (20.0) | 1 (10.0) |  |  |
| **Sleep Disturbance** |  |  |  | 2.24 | 0.135 |
| No difficulty, *N (%)* | 1 (6.7) | 0 (0.0) | 1 (10.0) |  |  |
| Little difficulty, *N (%)* | 11 (73.3) | 3 (60.0) | 8 (80.0) |  |  |
| Moderate difficulty, *N (%)* | 2 (13.3) | 1 (20.0) | 1 (10.0) |  |  |
| Severe difficulty, *N (%)* | 1 (6.7) | 1 (20.0) | 0 (0.0) |  |  |
| **Sleep Latency** |  |  |  | 0.50 | 0.480 |
| No difficulty, *N (%)* | 4 (26.7) | 1 (20.0) | 3 (30.0) |  |  |
| Little difficulty, *N (%)* | 6 (40.0) | 2 (40.0) | 4 (40.0) |  |  |
| Moderate difficulty, *N (%)* | 2 (13.3) | 0 (0.0) | 2 (20.0) |  |  |
| Severe difficulty, *N (%)* | 3 (20.0) | 2 (20.0) | 1 (10.0) |  |  |
| **Day Dysfunction due to Sleepiness** |  |  |  | 0.32 | 0.572 |
| No difficulty, *N (%)* | 5 (33.3) | 2 (40.0) | 3 (30.0) |  |  |
| Little difficulty, *N (%)* | 9 (60.0) | 3 (60.0) | 6 (60.0) |  |  |
| Moderate difficulty, *N (%)* | 1 (6.7) | 0 (0.0) | 1 (10.0) |  |  |
| Severe difficulty, *N (%)* | 0 (0.0) | 0 (0.0) | 0 (0.0) |  |  |
| **Sleep Efficiency** |  |  |  | 1.01 | 0.316 |
| No difficulty, *N (%)* | 8 (53.3) | 2 (40.0) | 6 (60.0) |  |  |
| Little difficulty, *N (%)* | 2 (13.3) | 0 (0.0) | 2 (20.0) |  |  |
| Moderate difficulty, *N (%)* | 3 (20.0) | 2 (40.0) | 1 (10.0) |  |  |
| Severe difficulty, *N (%)* | 2 (13.3) | 0 (20.0) | 1 (10.0) |  |  |
| **Overall Sleep Quality** |  |  |  | 0.79 | 0.375 |
| No difficulty, *N (%)* | 4 (26.7) | 1 (20.0) | 3 (30.0) |  |  |
| Little difficulty, *N (%)* | 10 (66.7) | 3 (60.0) | 7 (70.0) |  |  |
| Moderate difficulty, *N (%)* | 0 (0.0) | 0 (0.0) | 0 (0.0) |  |  |
| Severe difficulty, *N (%)* | 1 (6.7) | 1 (20.0) | 0 (0.0) |  |  |
| **Need Medication to Sleep** |  |  |  | 0.27 | 0.604 |
| No difficulty, *N (%)* | 13 (86.7) | 4 (80.0) | 9 (90.0) |  |  |
| Little difficulty, *N (%)* | 2 (13.3) | 1 (20.0) | 1 (10.0) |  |  |
| Moderate difficulty, *N (%)* | 0 (0.0) | 0 (0.0) | 0 (0.0) |  |  |
| Severe difficulty, *N (%)* | 0 (0.0) | 0 (0.0) | 0 (0.0) |  |  |
| **Perceived Cohesion Scale** | 4.71 (1.99) | 4.93 (0.77) | 4.60 (2.41) | 0.92 | 0.338 |
| Belonging Factor, *Mean (SD)* | 4.73 (2.02) | 5.00 (0.71) | 4.60 (2.46) | 0.85 | 0.356 |
| Morale Factor, *Mean (SD)* | 4.69 (1.96) | 4.87 (0.84) | 4.60 (2.37) | 0.92 | 0.338 |
|  |  |  |  |  |  |
|  |  |  |  |  |  |
