# Supplementary material for: Psychological well-being of healthcare workers during COVID-19 in a mental health institution
Source: PLoS One. 2024 Mar 18;19(3):e0300329. doi: 10.1371/journal.pone.0300329 (PMC10947715; doi:10.1371/journal.pone.0300329)
Supplement: S5 Table — (DOCX) [file pone.0300329.s005.docx]

**Supporting Information**

**Table 5**

Correlation between Time 1 Brief-COPE with Time point 1 Psychosocial variable- PSQI (n= 37)

|  | 1 | 2 | 3 | 4 | 5 | 6 | 7 | 8 |
| --- | --- | --- | --- | --- | --- | --- | --- | --- |
|  |  |  |  |  |  |  |  |  |
| **Problem-Focused Coping** | -0.056 | -0.144 | 0.049 | -0.030 | 0.079 | -0.200 | 0.034 | -0.032 |
| Active Coping | 0.042 | -0.273 | -0.029 | -0.046 | 0.145 | -0.231 | 0.016 | -0.051 |
| Use of Informational Support | -0.153 | -0.020 | 0.063 | 0.038 | 0.113 | -0.101 | -0.153 | 0.016 |
| Positive Reframing | -0.111 | 0.068 | 0.016 | 0.013 | 0.052 | -0.210 | 0.036 | -0.030 |
| Planning | -0.038 | -0.103 | 0.217 | -0.041 | -0.007 | -0.149 | 0.186 | 0.010 |
| **Emotion-Focused Coping** | 0.034 | 0.073 | 0.117 | 0.057 | 0.074 | -0.045 | 0.149 | 0.136 |
| Emotional Support | 0.016 | -0.009 | 0.103 | 0.113 | 0.295 | -0.056 | 0.033 | 0.166 |
| Venting | 0.000 | 0.089 | 0.008 | 0.197 | -0.061 | -0.002 | 0.139 | 0.079 |
| Humor | 0.102 | 0.256 | 0.032 | 0.216 | 0.098 | 0.054 | 0.081 | 0.207 |
| Acceptance | 0.079 | -0.049 | 0.066 | -0.151 | -0.102 | -0.092 | 0.189 | -0.019 |
| Religion | -0.028 | 0.060 | 0.161 | -0.116 | 0.092 | -0.036 | 0.016 | 0.088 |
| Self-blame | -0.135 | 0.119 | 0.040 | 0.252 | -0.079 | -0.010 | 0.151 | 0.035 |
| **Avoidant Coping** | 0.062 | 0.115 | 0.077 | 0.105 | 0.107 | -0.297 | 0.148 | 0.096 |
| Self-distraction | -0.026 | 0.042 | 0.069 | 0.105 | 0.044 | -0.306 | 0.064 | 0.070 |
| Denial | 0.086 | -0.015 | -0.268 | -0.044 | -0.036 | -0.102 | 0.139 | -0.114 |
| Substance use | - | - | - | - | - | - | - | - |
| Behavioral Disengagement | 0.160 | 0.249 | 0.145 | 0.129 | -0.004 | 0.227 | -0.165 | 0.143 |
|  |  |  |  |  |  |  |  |  |

1: Duration of Sleep; 2: Sleep Disturbance; 3: Sleep Latency; 4: Day Dysfunction due to Sleepiness; 5: Sleep Efficiency; 6: Overall Sleep Quality; 7: Need Medication to Sleep; 8: PSQI Total. Reported correlation is significant at the *p<0.017 level.
