# Supplementary material for: Psychological well-being of healthcare workers during COVID-19 in a mental health institution
Source: PLoS One. 2024 Mar 18;19(3):e0300329. doi: 10.1371/journal.pone.0300329 (PMC10947715; doi:10.1371/journal.pone.0300329)
Supplement: S6 Table — (DOCX) [file pone.0300329.s006.docx]

**Supporting Information**

**Table 6**

Correlation between Time 1 Brief-COPE with Time point 3 Psychosocial variables- DASS-21 & SWEMWBS (n= 15)

|  | 1 | 2 | 3 | 4 | 5 |
| --- | --- | --- | --- | --- | --- |
|  |  |  |  |  |  |
| **Problem-Focused Coping** | 0.058 | 0.080 | 0.058 | -0.344 | -0.119 |
| Active Coping | -0.082 | -0.034 | -0.082 | -0.418 | -0.080 |
| Use of Informational Support | 0.146 | 0.103 | 0.146 | -0.184 | -0.278 |
| Positive Reframing | 0.168 | 0.313 | 0.168 | -0.100 | 0.023 |
| Planning | 0.042 | 0.034 | 0.042 | -0.312 | 0.012 |
| **Emotion-Focused Coping** | 0.000 | -0.048 | 0.000 | -0.211 | -0.129 |
| Emotional Support | 0.063 | -0.052 | 0.063 | -0.259 | -0.271 |
| Venting | 0.233 | 0.245 | 0.233 | -0.461 | -0.200 |
| Humor | -0.381 | -0.256 | -0.381 | 0.065 | -0.040 |
| Acceptance | -0.145 | -0.020 | -0.145 | 0.099 | 0.215 |
| Religion | 0.123 | -0.051 | 0.123 | -0.042 | 0.057 |
| Self-blame | 0.350 | 0.237 | 0.350 | -0.065 | -0.442 |
| **Avoidant Coping** | 0.040 | 0.084 | 0.040 | -0.262 | 0.135 |
| Self-distraction | -0.021 | 0.121 | -0.021 | -0.171 | -0.105 |
| Denial | 0.155 | 0.043 | 0.155 | -0.316 | 0.330 |
| Substance use | - | - | - | - | - |
| Behavioral Disengagement | -0.105 | -0.130 | -0.105 | -0.464 | -0.087 |
|  |  |  |  |  |  |

1: DASS-21 Depression; 2: DASS-21 Anxiety; 3: DASS-21 Stress; 4: SWEMWBS level of well-being, 5: Perceived Cohesion Scale. Reported correlation is significant at the *p<0.017 level.
