# Supplementary material for: Psychological well-being of healthcare workers during COVID-19 in a mental health institution
Source: PLoS One. 2024 Mar 18;19(3):e0300329. doi: 10.1371/journal.pone.0300329 (PMC10947715; doi:10.1371/journal.pone.0300329)
Supplement: S7 Table — (DOCX) [file pone.0300329.s007.docx]

**Supporting Information**

**Table 7**

Correlation between Time 1 Brief-COPE with Time point 3 Psychosocial variable- PSQI (n= 15)

|  | 1 | 2 | 3 | 4 | 5 | 6 | 7 | 8 |
| --- | --- | --- | --- | --- | --- | --- | --- | --- |
|  |  |  |  |  |  |  |  |  |
| **Problem-Focused Coping** | -0.192 | -0.918 | 0.000 | -0.206 | -0.023 | -0.202 | -0.272 | -0.190 |
| Active Coping | -0.214 | -0.238 | -0.035 | -0.163 | -0.037 | -0.128 | -0.164 | -0.169 |
| Use of Informational Support | -0.097 | 0.061 | 0.130 | -0.276 | -0.100 | -0.130 | -0.249 | -0.107 |
| Positive Reframing | -0.147 | -0.306 | 0.060 | -0.098 | -0.265 | 0.029 | -0.357 | -0.173 |
| Planning | -0.435 | -0.409 | 0.273 | -0.193 | -0.050 | -0.505 | -0.291 | -0.385 |
| **Emotion-Focused Coping** | -0.337 | -0.296 | -0.198 | -0.154 | 0.012 | -0.375 | -0.155 | -0.299 |
| Emotional Support | -0.123 | -0.015 | 0.024 | -0.084 | -0.013 | -0.103 | -0.106 | -0.185 |
| Venting | -0.160 | -0.154 | -0.096 | -0.084 | -0.165 | -0.044 | -0.233 | -0.207 |
| Humor | -0.263 | -0.260 | -0.136 | -0.063 | -0.129 | -0.198 | 0.119 | -0.282 |
| Acceptance | -0.309 | -0.510 | -0.110 | -0.321 | -0.102 | -0.335 | -0.290 | -0.261 |
| Religion | -0.334 | -0.270 | -0.223 | 0.000 | 0.198 | -0.428 | 0.103 | -0.244 |
| Self-blame | 0.277 | 0.278 | 0.254 | -0.021 | 0.019 | 0.177 | -0.191 | 0.164 |
| **Avoidant Coping** | -0.270 | -0.162 | -0.219 | -0.269 | 0.146 | -0.351 | -0.020 | -0.240 |
| Self-distraction | -0.012 | 0.168 | 0.060 | -0.333 | 0.050 | -0.174 | 0.063 | -0.043 |
| Denial | -0.329 | -0.488 | -0.543 | 0.085 | -0.046 | -0.268 | -0.258 | -0.385 |
| Substance use | - | - | - | - | - | - | - | - |
| Behavioral Disengagement | -0.244 | -0.076 | -0.329 | -0.348 | 0.409 | -0.400 | -0.105 | -0.135 |
|  |  |  |  |  |  |  |  |  |

1: Duration of Sleep; 2: Sleep Disturbance; 3: Sleep Latency; 4: Day Dysfunction due to Sleepiness; 5: Sleep Efficiency; 6: Overall Sleep Quality; 7: Need Medication to Sleep; 8: PSQI Total. Reported correlation is significant at the *p<0.017 level.
