# Supplementary material for: Psychological well-being of healthcare workers during COVID-19 in a mental health institution
Source: PLoS One. 2024 Mar 18;19(3):e0300329. doi: 10.1371/journal.pone.0300329 (PMC10947715; doi:10.1371/journal.pone.0300329)
Supplement: S8 Table — (DOCX) [file pone.0300329.s008.docx]

**Supporting Information**

**Table 8**

*Q.5 Do you have any thoughts on the areas for improvement in IMH's response in managing the COVID-19 cases*

| **Category** | **Theme** | **Illustrative Example** |
| --- | --- | --- |
| Areas done well | Teamwork and camaraderie (21) | *Like certain things need to be done immediately one ah, they (peers) will like come together and help.*  *Teamwork is still there, still going strong.* |
|  | Prompt actions to contain infection (2) | *the management… got the ward for the COVID positive patient, … isolate them in one ward so won’t go to other wards*  *immediately give the protocol like how to do, how to manage, where to send, who to call so I feel it’s sufficient la* |
|  | Clear and prompt updates from management (2) | *(the team) will like come together and help.* |
|  | Clear and prompt updates within teams (2) | *…* *printout, TigerText, then ah verbal updates when we have others. Then those not on shift, we keep them updated also like group message and all that…* |
|  | Efficiency in responding to feedback and requests | *immediately give the protocol like how to do, how to manage, where to send…* |
|  | Psychological safety | *Can freely text them (Immediate work supervisors) to ask for latest update (no restriction)* |
|  | Appreciation | *(Immediate work supervisors) still treating us even though we can buy food already* |
|  |  |  |
| Areas for improvement | Management of visitors | *Visitors that managed to sneak into the ward and ward staff have to handle them (additional work) despite going through screening centre (pretended they don't know the restrictions)*  *Our staff need to contact 40 relatives, besides spending time on the ground and handling difficult pts, they still have to call* |
|  |  |  |
|  | Rapidly changing instructions | *instructions keep on revising every hour that kind of thing*  *It’ll be best like you know if they have a standardized management for these COVID patients as in not to always like change the guidelines because like for us the ground staff, we will also be very confused in nursing these patients la. Then 24/7 we will have to call IFC to confirm, so it’ll be best to have a standardized guideline from MOH to us.* |
|  | Making Difficulty Decisions | *Not many people will … like voluntarily want to go like go to COVID ward and look after the COVID patients. So not that really very easy for us like to nominate one to be deployed to the COVID ward at the last minute so this kind of arrangement and like should be done early* |
|  | Physical Working Environment | *I hope there's aircons everywhere. Because it’s really hot, even without full PPE wearing the N95. One more thing is that the place for us to rest is not enough* |
|  | Early Warning | *Management can alert us prior the outbreak even though its already happened in other countries, … we can mentally prepare ourselves first* |
|  | Future Isolation Wards | *have standby wards for this kind of outbreak instead of converting existing wards to isolation wards (should be done beforehand)* |
